# Supplementary material for: A GREB1-steroid receptor feedforward mechanism governs differential GREB1 action in endometrial function and endometriosis
Source: Nat Commun. 2024 Mar 2;15:1947. doi: 10.1038/s41467-024-46180-4 (PMC10908778; doi:10.1038/s41467-024-46180-4)
Supplement: Supplementary file 1 — Supplementary Information [file 41467_2024_46180_MOESM1_ESM.pdf]

Supplementary Materials for

A GREB1-steroid receptor feedforward mechanism governs differential GREB1 action in endometrial function and endometriosis

Sangappa B. Chadchan[1], Pooja Popli[1], Zian Liao[1], Eryk Andreas[2], Michelle Dias[3], Tianyuan Wang[4], Stephanie J. Gunderson[2], Patricia T. Jimenez[2], Denise G. Lanza[5], Rainer B. Lanz[6], Charles E. Foulds[7], Diana Monsiavais[1], Francesco J. DeMayo[8], Hari Krishna Yalamanchili[3,9,10], Emily S. Jungheim[2,11], Jason D. Heaney[5], John P. Lydon[6], Kelle H. Moley[2], Bert W. O'Malley[6], and Ramakrishna Kommagani[1,12*]

The PDF file includes:

Supplementary Fig. 1 GREB1 acts as a PR cofactor in human endometrial stromal cells.

Supplementary Fig. 2 *Greb1* KO mice have normal ovarian function.

Supplementary Fig. 3 Compromised progesterone signaling in the uteri of *Greb1* KO mice.

Supplementary Fig. 4 Transcript levels of *Igf1, Mcm2, Klf4,* and *Klf15* in uteri of mimicked hormonal states of pregnancy.

Supplementary Fig. 5 Decidualization is defective in *Greb1* KO mice.

Supplementary Fig. 6 Estradiol (E2) levels are unaltered in serum collected from control or *Greb1* endometriotic females.

Supplementary Table 1 List of primers and TaqMan probes

Supplementary Table 2 List of antibodies

Supplementary Table 3 List of Primers used for Chromatin Immunoprecipitation (ChIP).

Supplementary figure legends

Supplementary Fig. 1 GREB1 acts as a PR cofactor in human endometrial stromal cells. a, Visualization of GREB1 and PR ChIP-seq peaks enriched around the enhancer region of FOXO1. **b**, Gene ontology of enriched signaling pathways from GREB1 HES cistrome.

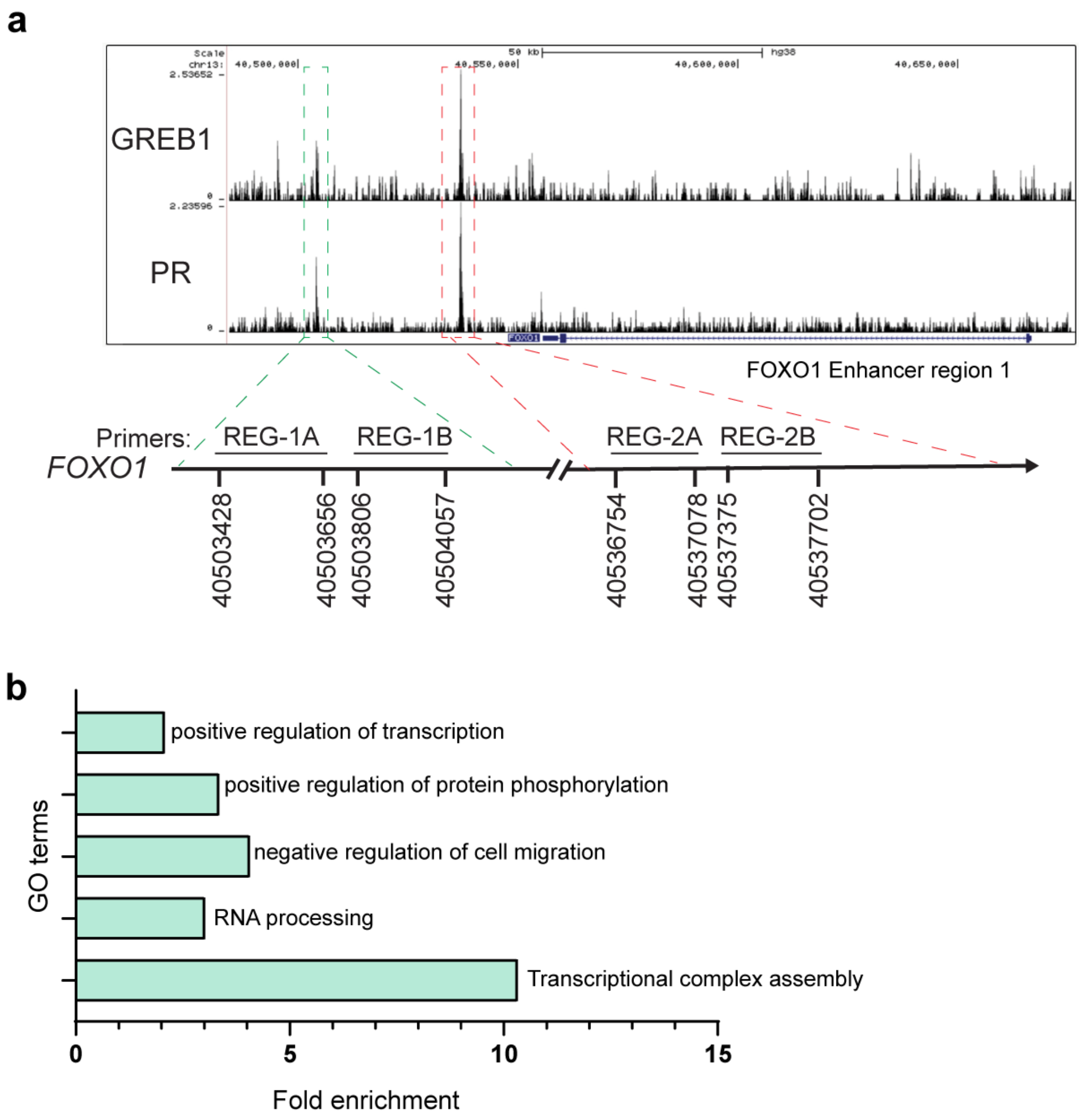

Supplementary Fig. 2 *Greb1* KO mice have normal ovarian function. a, Graph depicting the number of litter per female from wild type (n=9) and Greb1 KO (n=8) in six-month fertility tests. Paired, two-tailed, t-test. Data reported as the mean ± SEM. *P< 0.05, ***P< 0.001, ****P< 0.0001 **b**, Analysis of GREB1 transcript and protein levels in ovaries from wild-type (WT) (n=6) and *Greb1* KO mice (n=6). Paired, two-tailed, t-test. Data reported as the mean ± SEM. *P< 0.05, ***P< 0.001, ****P< 0.0001. Red arrowhead indicates the GREB1-positive cells and black arrowhead indicates GREB1-negative cells; scale bar: 200 μm. **c**, The relative mRNA concentration of *Esr1* and *Pgr* (determined by QRT-PCR) in the ovary of WT (n=6) and *Greb1* KO mice (n=6). Paired, two-tailed, t-test. Data reported as the mean ± SEM. *P< 0.05, ***P< 0.001, ****P< 0.0001. **d**, Representative cross-sectional images of the ovary from WT and *Greb1* KO mice stained for ER-alpha and PR; scale bar: 200 μm. At the time of tissue collection, most of the females from WT or *Greb1* KO were at the diestrus or metestrus stage of the estrous cycle.

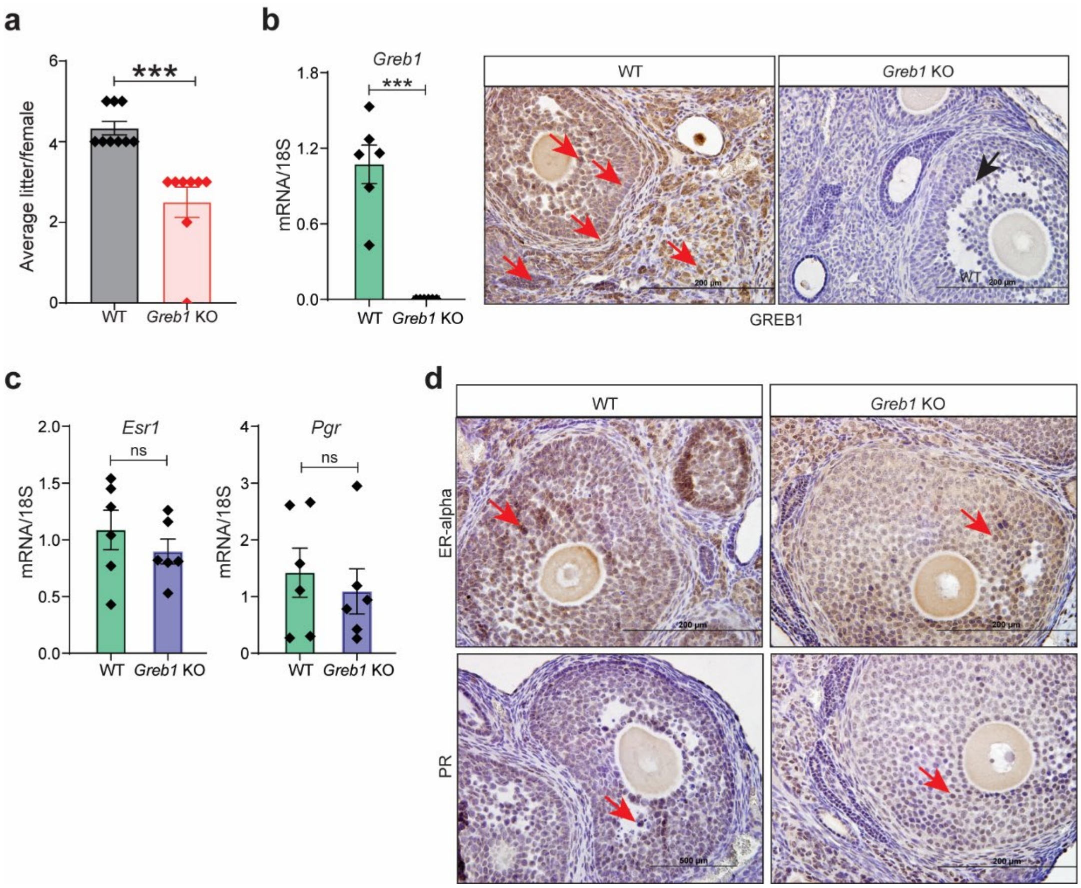

Supplementary Fig. 3 Compromised progesterone signaling in the uteri of *Greb1* KO mice. **a**, Relative transcript levels of *Muc1* in uterine tissues of dpc 4 from wild type (n=5) and *Greb1* KO mice (n=5). Paired, two-tailed, t-test. Data reported as the mean ± SEM. *P< 0.05, ***P< 0.001, ****P< 0.0001. **b**, Relative mRNA levels of *Greb1, Pgr,* and *Esr1* in the uteri of *Greb1* KO and WT mice at dpc 4 (n=5 for each genotype). Paired, two-tailed, t-test. Data reported as the mean ± SEM. *P< 0.05, ***P< 0.001, ****P< 0.0001. **b**, Relative mRNA levels of progesterone target genes*, Foxo1, Areg,* and *Hand2* in the uteri of *Greb1* KO and WT mice at dpc 4 (n=5 for each genotype). Paired, two-tailed, t-test. Data reported as the mean ± SEM. *P< 0.05, ***P< 0.001, ****P< 0.0001.

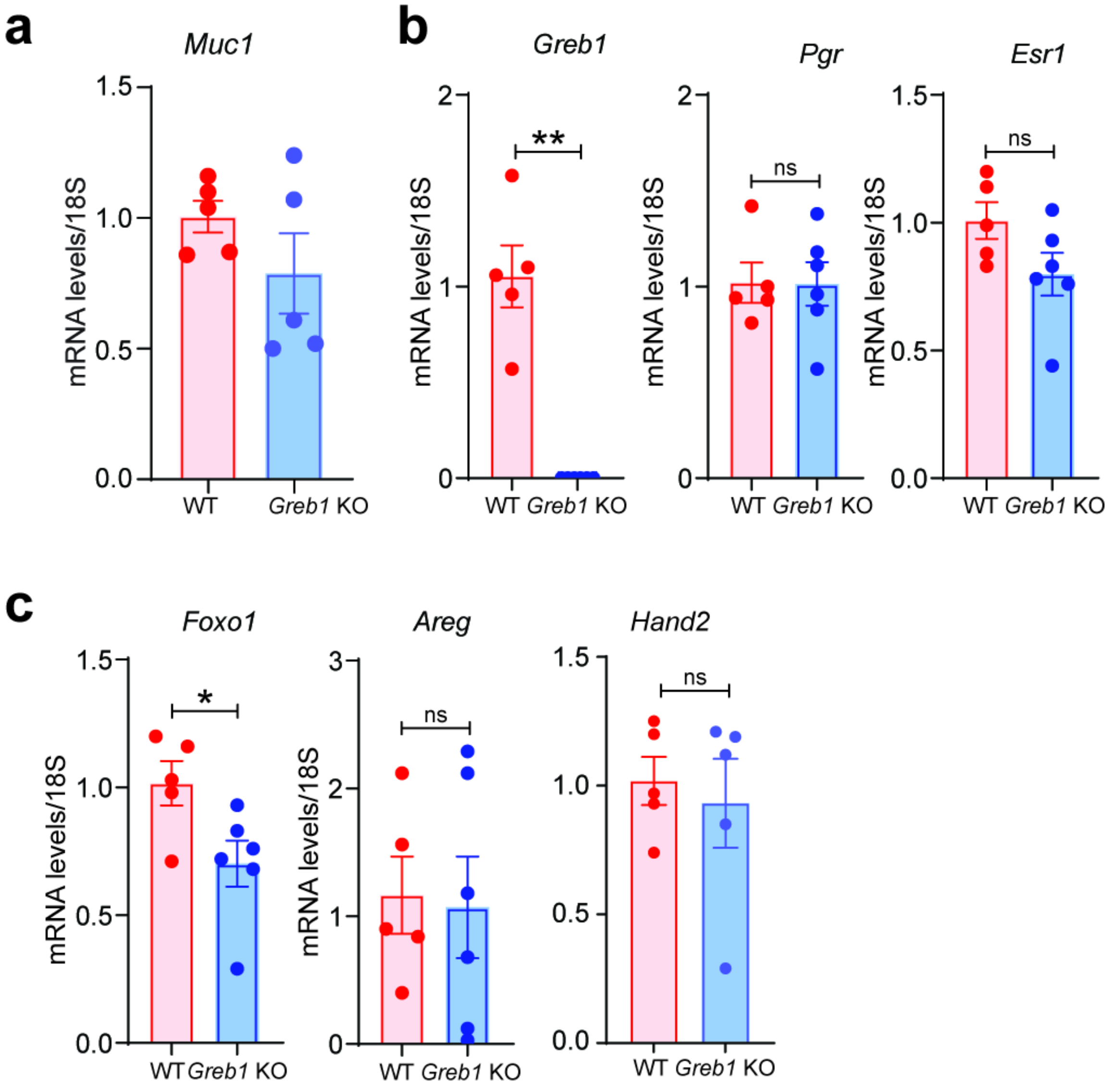

Supplementary Fig. 4 Transcript levels of *Igf1, Mcm2, Klf4,* and *Klf15* in uteri of mimicked hormonal states of pregnancy. a-d, Relative mRNA levels of *Igf1* **a**, *Mcm2* **b**, *Klf4* **c**, and *Klf15* **d**, from uteri from WT and *Greb1* KO mice in the indicated treatment groups. Data was analyzed by one-way ANOVA with Tukey's multiple comparisons post-test and presented as mean ± SEM (n =4-6), $P< 0.05$, $^{**}P<0.01$, $^{***}P< 0.001$, and ns, non-significant.

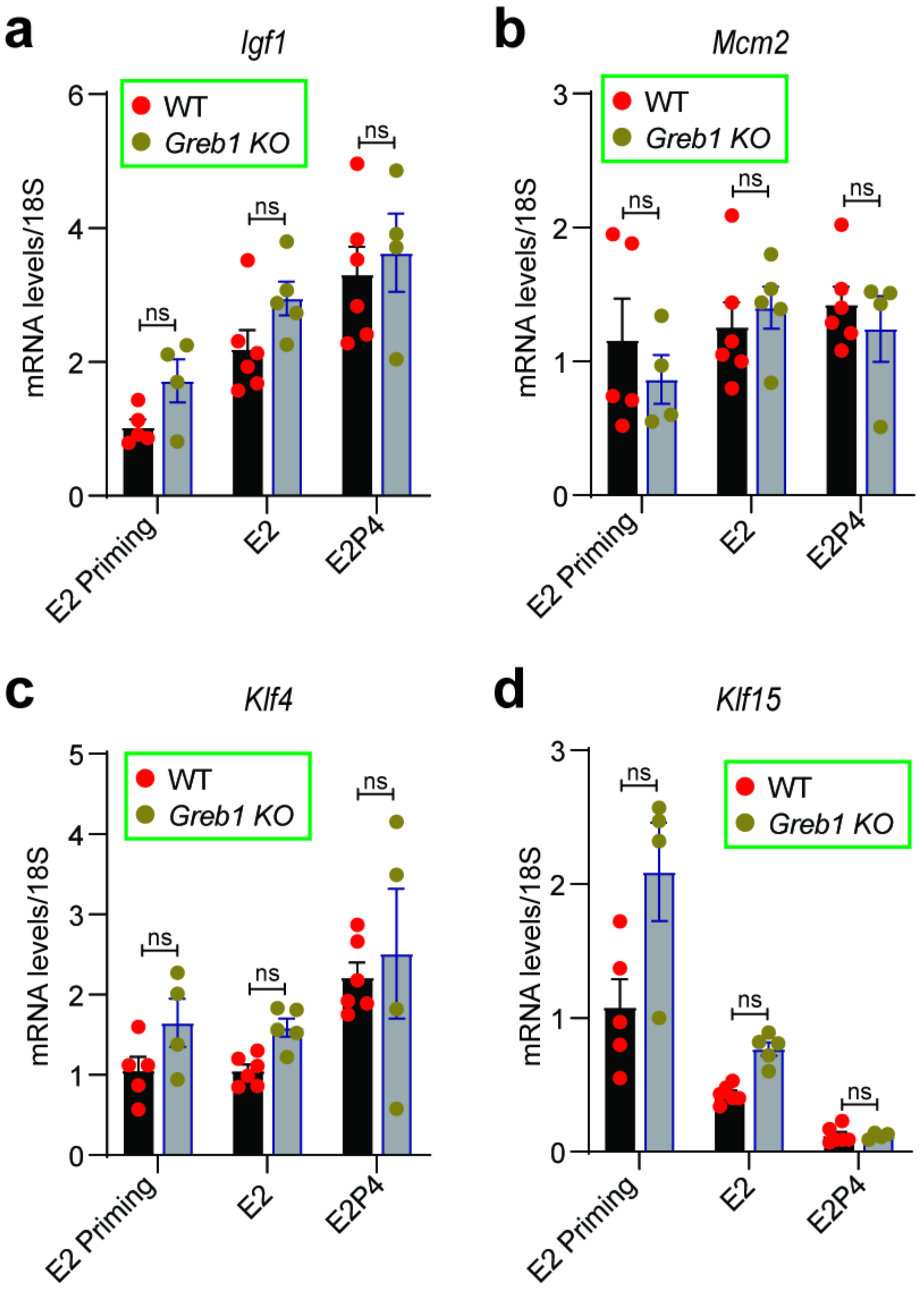

Supplementary Fig. 5 Decidualization is defective in *Greb1* KO mice. a, Timeline for induction of artificial decidualization in ovariectomized mice. **b**, Representative images showing the gross morphology of C: control and S: stimulated (oil-treated) uterine horns from WT and *Greb1* KO mice collected 2 days after oil injection. The ratio between the wet weight of the oil-treated horn to the wet weight of the untreated horn was calculated for each WT and *Greb1* KO (n = 5-6) mouse; then ratios were averaged and compared across genotypes by using Paired, two-tailed, t-test. Data reported as the mean ± SEM. *P< 0.05, ***P< 0.001, ****P< 0.0001. **c**, Representative cross-sectional images of the oil-treated uterus stained for Phospho-Histone H3 (PH3) from WT and *Greb1* KO mice. Red arrowhead indicates the PH3-positive cells and black arrowhead indicates PH3-negative cells; scale bar: 200 µm. The graph on the right depicts the percentage of ESCs (Endometrial stromal cells) undergoing proliferation in the stimulated horn of the WT (n=6) and *Greb1* KO (n=5) mice two days following receipt of the deciduogenic stimulus. Percentage of PH3 positive cells was calculated by counting positive cells in four separate fields at 400X magnification in uteri by an investigator blinded to treatment groups and plotted as percent positive cells relative to total cells. Paired, two-tailed, t-test. Data reported as the mean ± SEM. *P< 0.05, ***P< 0.001, ****P< 0.0001. **d**, Relative mRNA levels of decidualization marker genes (*Bmp2* and *Wnt4*) assayed by qRT-PCR. Data was analyzed by one-way ANOVA with Tukey's multiple comparisons post-test and presented as mean ± SEM (n=4-6), P< 0.05, **P<0.01, ***P< 0.001, and ns, non-significant.

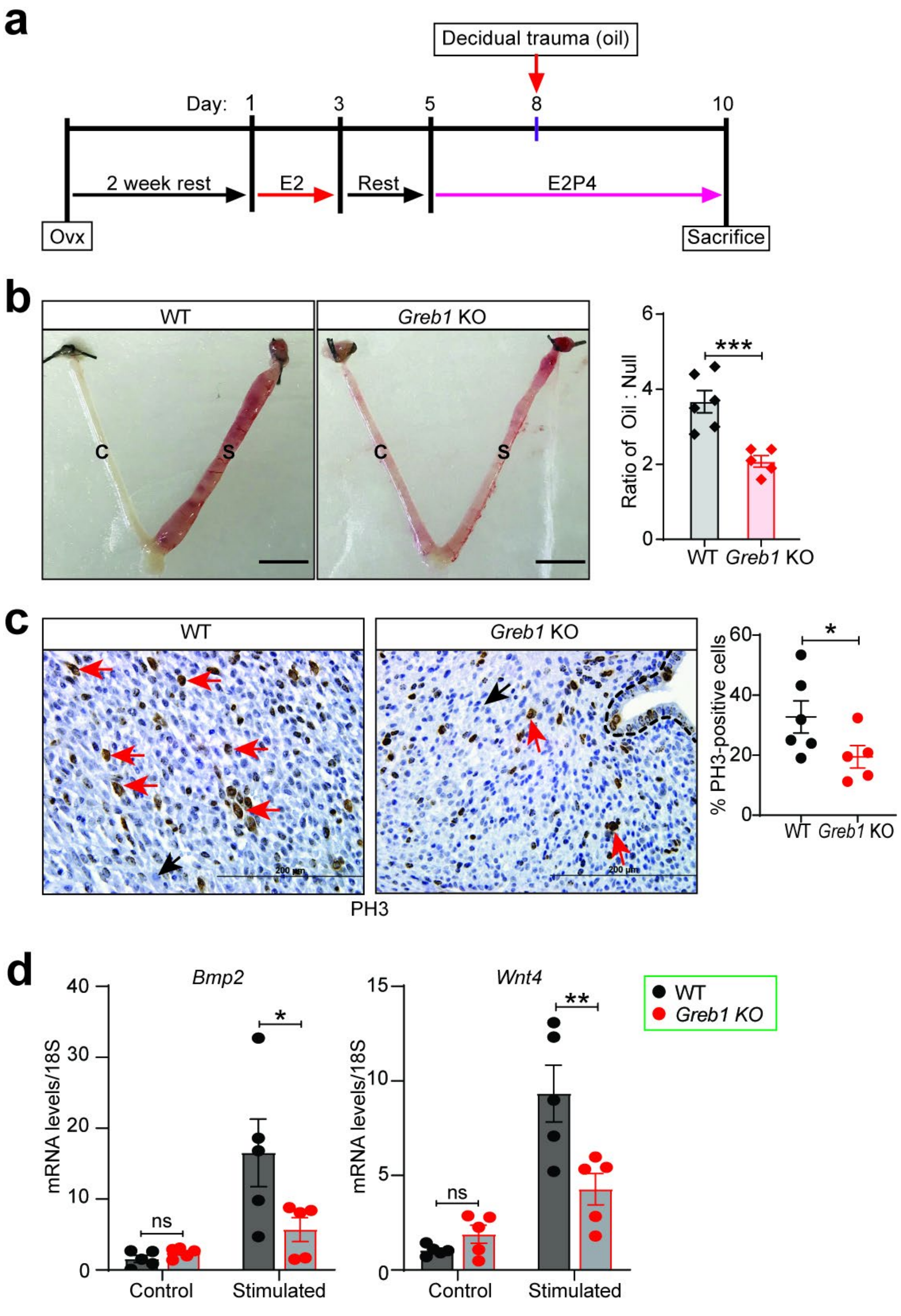
a
Decidual trauma (oil)
Day: 1 3 5 8 10
2 week rest
E2
Rest
E2P4
Ovx
Sacrifice
b
WT
Greb1 KO
C
S
Ratio of Oil : Null
***
WT Greb1 KO
c
WT
Greb1 KO
% PH3-positive cells
*
WT Greb1 KO
PH3
d
Bmp2
Wnt4
mRNA levels/18S
ns
*
**
Control
Stimulated
WT
Greb1 KO

Supplementary Fig. 6 Estradiol (E2) levels are unaltered in serum collected from control or *Greb1* endometriotic females. Graphs depicting the level of estrogen from WT (n=5) or *Greb1* endometriotic KO (n=5) mice. Paired, two-tailed, t-test. Data reported as the mean ± SEM. *P< 0.05, ***P< 0.001, ****P< 0.0001.

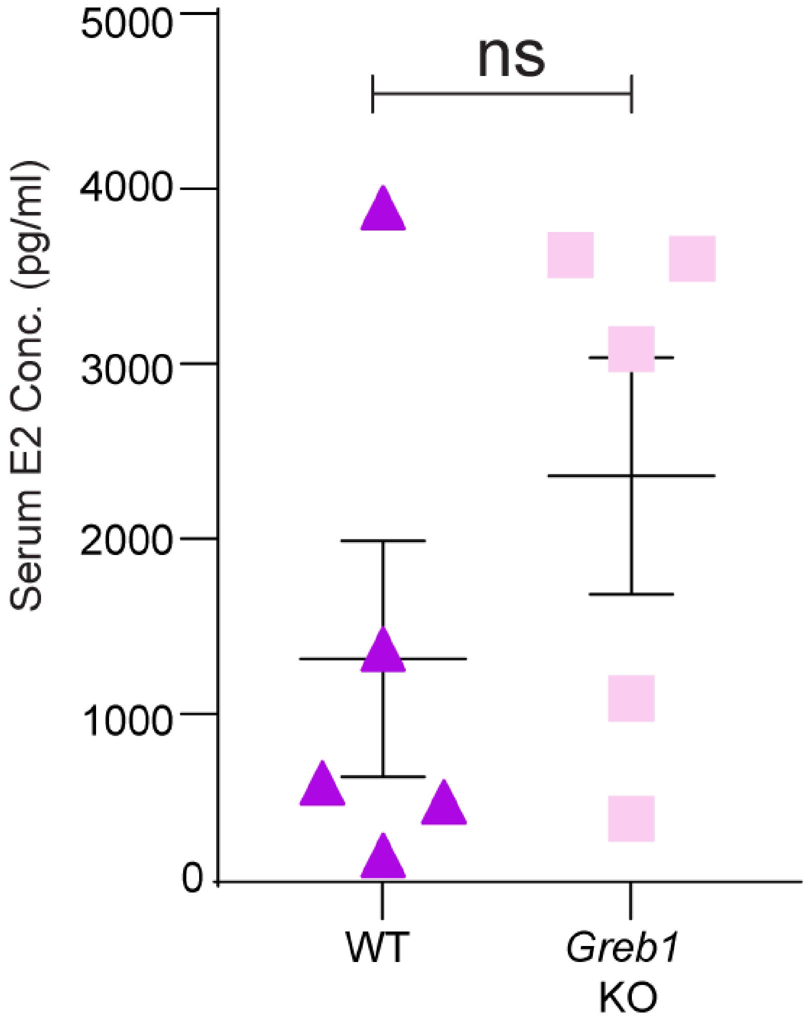

Supplementary Table 1 List of primers and TaqMan probes

Gene name	Species	Application, Chemistry	Company	Sequence/Cat. No.
Greb1 KO	Mouse	Genotyping	IDT	P1: GACAGGGTGTTTCCTTTTGC<br>P2: TTAGGCCACCATTGGAAACT<br>P3: ACAACCTCAGGCTGCAATTT
Greb1	Mouse	qPCR, TaqMan	ABI	Mm00479259_m1
Esr1	Mouse	qPCR, TaqMan	ABI	Mm00433149_m1
Pgr	Mouse	qPCR, TaqMan	ABI	Mm00435628_m1
Fgf18	Mouse	qPCR, TaqMan	ABI	Mm00433286_m1
Areg	Mouse	qPCR, TaqMan	ABI	Mm01354339_m1
Bmp2	Mouse	qPCR, TaqMan	ABI	Mm01340178_m1
Igf1	Mouse	qPCR, TaqMan	ABI	Mm00439560_m1
Klf4	Mouse	qPCR, TaqMan	ABI	Mm00516104_m1
Klf15	Mouse	qPCR, TaqMan	ABI	Mm00517792_m1
Ccnd1	Mouse	qPCR, TaqMan	ABI	Mm00432359_m1
Mcm2	Mouse	qPCR, TaqMan	ABI	Mm00484804_m1
Wnt4	Mouse	qPCR, TaqMan	ABI	Mm01194003_m1
Ihh	Mouse	qPCR, TaqMan	ABI	Mm00439613_m1
Foxo1	Mouse	qPCR, TaqMan	ABI	Mm00490671_m1
Cyp26a1	Mouse	qPCR, TaqMan	ABI	Mm00514486_m1
Il13ra2	Mouse	qPCR, TaqMan	ABI	Mm00515166_m1
Muc1	Mouse	qPCR, TaqMan	ABI	Mm00449604_m1
Hand2	Mouse	qPCR, TaqMan	ABI	Mm00439247_m1
GREB1	Human	qPCR, TaqMan	ABI	Hs00536409_m1
FOXO1	Human	qPCR, TaqMan	ABI	Hs00231106_m1
CCND1	Human	qPCR, TaqMan	ABI	Hs00765553_m1
IGF1	Human	qPCR, TaqMan	ABI	Hs01547656_m1
ESR1	Human	qPCR, TaqMan	ABI	Hs01046816_m1
18S	Human<br>Mouse	qPCR, TaqMan	ABI	4318839

*All primer sequences are written 5’ to 3’

ABI-applied biosystems

IDT-integrated DNA technologies.

Supplementary Table 2 List of antibodies

Antibody	Company, Catalogue number	Application and Titer
GREB1	Abcam, ab72999	Immunoblotting (1:1000), ChIP (3 ug)
GREB1	ThermoFisher Scientific PA5-50673	Immunohistochemistry (1:100)<br>Immunofluorescence (1:100)
Ki-67	Abcam, ab15580	Immunohistochemistry (1:100)
ER-α	Abcam, ab75635	Immunohistochemistry (1:100)
Cyclin D1	CST, #55506	Immunohistochemistry (1:100)
Phospho-Histone H3	Millipore Sigma, 06-570	Immunohistochemistry<br>Immunofluorescence (1:100)
MUC1	Abcam, ab15481	Immunofluorescence (1:100)
PR Antibody (F-4) AC	SCBT, sc-166169 AC	Immunoprecipitation (3 ug)
normal mouse IgG-AC	SCBT, sc-2343	Immunoprecipitation (3 ug)
PR	SCBT, PR (H-190) sc-7208	Immunohistochemistry (1:100)<br>Immunoblotting (1:1000)<br>ChIP (3 ug)
Normal Rabbit IgG	CST, #2729	Immunohistochemistry (1:1000)<br>ChIP (3 ug)
Goat anti-Rabbit IgG (H+L)	ThermoFisher Scientific, A32731	Immunofluorescence (1:100)
GAPDH	CST, #2118S	Immunoblotting (1:5000)
Anti-rabbit IgG, HRP-linked	CST, #7074	Immunoblotting (1:3000)

Supplementary Table 3 List of Primers used for Chromatin Immunoprecipitation (ChIP)

Primers	Company	Sequence
FOXO1 Region 1A Forward	Sigma-Aldrich	GGTCAACCAATGCACACAATG
FOXO1 Region 1A Reverse	Sigma-Aldrich	CGTAGGTCCCTGGATGAGTATAA
FOXO1 Region 1B Forward	Sigma-Aldrich	CAATGCCGACGTCCAGAG
FOXO1 Region 1B Reverse	Sigma-Aldrich	GGGATTCCTGAGAACACTAAGC
FOXO1 Region 2A Forward	Sigma-Aldrich	GCTCCCAAGAATCATGTGTTATG
FOXO1 Region 2A Reverse	Sigma-Aldrich	GTGTTTGTGAAGCAGGTTGG
FOXO1 Region 2B Forward	Sigma-Aldrich	GGAGCTGGTTCACAGAAAGT
FOXO1 Region 2B Reverse	Sigma-Aldrich	CCCATCAAAGTAATCAGGGACA
UNTR Forward	Sigma-Aldrich	CTGTACCTGGGGTTCATTCATT
UNTR Reverse	Sigma-Aldrich	CAGTAAGCCGTTCACTCTCACA

*All primer sequences are written 5’ to 3’
