# Supplementary material for: A GREB1-steroid receptor feedforward mechanism governs differential GREB1 action in endometrial function and endometriosis
Source: Nat Commun. 2024 Mar 2;15:1947. doi: 10.1038/s41467-024-46180-4 (PMC10908778; doi:10.1038/s41467-024-46180-4)
Supplement: Supplementary file 3 — Reporting Summary [file 41467_2024_46180_MOESM3_ESM.pdf]

Corresponding author(s): RAMAKRISHNA KOMMAGANI

Last updated by author(s): Jan 10, 2024

Reporting Summary

Nature Portfolio wishes to improve the reproducibility of the work that we publish. This form provides structure for consistency and transparency in reporting. For further information on Nature Portfolio policies, see our Editorial Policies and the Editorial Policy Checklist.

Statistics

For all statistical analyses, confirm that the following items are present in the figure legend, table legend, main text, or Methods section.

n/a	Confirmed	
☐	☒	The exact sample size (*n*) for each experimental group/condition, given as a discrete number and unit of measurement
☐	☒	A statement on whether measurements were taken from distinct samples or whether the same sample was measured repeatedly
☐	☒	The statistical test(s) used AND whether they are one- or two-sided<br>*Only common tests should be described solely by name; describe more complex techniques in the Methods section.*
☐	☒	A description of all covariates tested
☐	☒	A description of any assumptions or corrections, such as tests of normality and adjustment for multiple comparisons
☐	☒	A full description of the statistical parameters including central tendency (e.g. means) or other basic estimates (e.g. regression coefficient) AND variation (e.g. standard deviation) or associated estimates of uncertainty (e.g. confidence intervals)
☐	☒	For null hypothesis testing, the test statistic (e.g. *F*, *t*, *r*) with confidence intervals, effect sizes, degrees of freedom and *P* value noted<br>*Give P values as exact values whenever suitable.*
☒	☐	For Bayesian analysis, information on the choice of priors and Markov chain Monte Carlo settings
☒	☐	For hierarchical and complex designs, identification of the appropriate level for tests and full reporting of outcomes
☒	☐	Estimates of effect sizes (e.g. Cohen's *d*, Pearson's *r*), indicating how they were calculated

Our web collection on statistics for biologists contains articles on many of the points above.

Software and code

Policy information about availability of computer code

Data collection	*No software wwas used for data collection*
Data analysis	No custom alogrithms or software were used for this study. All software used were publically available, which were outlined in method sections

For manuscripts utilizing custom algorithms or software that are central to the research but not yet described in published literature, software must be made available to editors and reviewers. We strongly encourage code deposition in a community repository (e.g. GitHub). See the Nature Portfolio guidelines for submitting code & software for further information.

Data

Policy information about availability of data

All manuscripts must include a data availability statement. This statement should provide the following information, where applicable:

- Accession codes, unique identifiers, or web links for publicly available datasets
- A description of any restrictions on data availability
- For clinical datasets or third party data, please ensure that the statement adheres to our policy

Cut&Run -sequencing datasets generated in this study have been deposited in the NCBI GEO database under accession code: GSE254175. Source data is provided with this paper.

Research involving human participants, their data, or biological material

Policy information about studies with human participants or human data. See also policy information about sex, gender (identity/presentation), and sexual orientation and race, ethnicity and racism.

Reporting on sex and gender	Human Female participants were not directly involved in this study. Only their biological material was used to obtain the cells. Study was done under the protocol (IRB ID # 201612127 and 201807160) approved by the Washington University in St. Louis School of Medicine Institutional Review Board in accordance with the criteria set by the Declaration of Helsinki.
Reporting on race, ethnicity, or other socially relevant groupings	NA
Population characteristics	NA
Recruitment	NA
Ethics oversight	NA

Note that full information on the approval of the study protocol must also be provided in the manuscript.

Field-specific reporting

Please select the one below that is the best fit for your research. If you are not sure, read the appropriate sections before making your selection.

☒ Life sciences ☐ Behavioural & social sciences ☐ Ecological, evolutionary & environmental sciences

For a reference copy of the document with all sections, see nature.com/documents/nr-reporting-summary-flat.pdf

Life sciences study design

All studies must disclose on these points even when the disclosure is negative.

Sample size	All the animal experiments were conducted using the sample size, minimum n=5 -10 per group based on our previous studies (PMCID: PMC5909854
Data exclusions	no data was excluded from the analysis
Replication	All experiments on primary cells obtained from human endometrial biopsies were repeated three times independently from different patients. We also used transformed cell lines IHEEC-luc cells and for that experiments were repeated indepnendtly across individual cell frozenstocks of different passages. All attempts at replication were successful and we ensure all experiments were reproducible across multiple biological replicates and between mutliple individual researchers
Randomization	Samples and organisms were randomly allocated to groups.
Blinding	Investigators were blinded to the treatment groups during data collection and subsequent data analysis.

Reporting for specific materials, systems and methods

We require information from authors about some types of materials, experimental systems and methods used in many studies. Here, indicate whether each material, system or method listed is relevant to your study. If you are not sure if a list item applies to your research, read the appropriate section before selecting a response.

Materials & experimental systems

n/a	Involved in the study	
☐	☒	Antibodies
☐	☒	Eukaryotic cell lines
☒	☐	Palaeontology and archaeology
☐	☒	Animals and other organisms
☒	☐	Clinical data
☒	☐	Dual use research of concern
☒	☐	Plants

Methods

n/a	Involved in the study	
☒	☐	ChIP-seq
☒	☐	Flow cytometry
☒	☐	MRI-based neuroimaging

Antibodies

Antibodies used	GREB1 (Abcam, ab72999 Immunoblotting, 1:1000; ChIP: 3 ug)<br>GREB1 (Thermofisher Scientific, PA5-50673, Immunohistochemistry, Immunofluorescence, 1:100)

Ki-67 Abcam, ab15580 Immunohistochemistry , 1:100
ER-α Abcam, ab75635 Immunohistochemistry , 1:100
Cyclin D1 CST, #55506 Immunohistochemistry , 1:100
Phospho-Histone H3 Millipore Sigma, 06-570 Immunohistochemistry
Immunofluorescence , 1:100
MUC1 Abcam, ab15481 Immunofluorescence , 1:100
PR Antibody (F-4) AC SCBT, sc-166169 AC Immunoprecipitation , 3 ug
normal mouse IgG-AC SCBT, sc-2343 Immunoprecipitation , 3 ug
PR SCBT, PR (H-190) sc-7208 Immunohistochemistry , 1:100
Immunoblotting , 1:1000
ChIP , 3 ug
Normal Rabbit IgG CST, #2729 Immunohistochemistry , 1:1000
ChIP , 3 ug
Goat anti-Rabbit IgG (H+L) Thermofisher Scientific, A32731 Immunofluorescence , 1:200
GAPDH CST, #2118S Immunoblotting , 1:5000
Anti-rabbit IgG, HRP-linked CST, #7074 Immunoblotting , 1:3000

Validation	All primary antibodies are validated for the detection of human or mouse antigen of interest according to manufacturer's websites

Eukaryotic cell lines

Policy information about cell lines and Sex and Gender in Research

Cell line source(s)	The primary cells (Human Endometriotic Stromal Cells) were isolated from human endometriotic lesions. All donors were female The iHEECs/Luc cell line were obtained from Dr. Sang Han Jun, Department of Molecular and Cellular Biology, Baylor College of Medicine, Houston, TX, 77030, USA.
Authentication	Authentication of cell lines was not performed by the authors.
Mycoplasma contamination	Cell lines were confirmed to be mycoplasma-free.
Commonly misidentified lines (See ICLAC register)	No commonly misidentified cell lines were used.

Animals and other research organisms

Policy information about studies involving animals; ARRIVE guidelines recommended for reporting animal research, and Sex and Gender in Research

Laboratory animals	Experimental control and GREB1 KO female mice from C57BL/6 background were used for this study. All experiments were initiated at 8-wee old age
Wild animals	This study did not involve wild animals
Reporting on sex	Since we focussed only on uterine functions , our study is limited to only females
Field-collected samples	This study did not involve samples collected in the field.
Ethics oversight	All animal studies were performed according to a protocol (number 20160227) approved by the Institutional Animal Care and Use Committee of Washington University School of Medicine, Saint Louis, MO, USA.

Note that full information on the approval of the study protocol must also be provided in the manuscript.

Plants

Seed stocks	NA
Novel plant genotypes	NA
Authentication	NA
